# The British Journals: Pathology, Practical Medicine, and Therapeutics

**Published:** 1842-01

**Authors:** 


### PATHOLOGY, PRACTICAL MEDICINE, AND THERAPEUTICS.

Antiquarian Notices of Leprosy and Leper Hospitals in Scotland and England. This an extremely elaborate and erudite paper to show the former frequency and severity of this loathsome disease in these kingdoms. According to the Anglo-Saxon lexicographies of Sommer, Lye, and Bosworth, "leprosy" was heretofore known by the singular and striking term "seo mycle adl," "the muckle or great evil" or disease. In the year 1200, there were in the counties of Northumberland, Cumberland, and Durham, a variety of hospitals, exclusively devoted to lepers. Three of these contained so many as ninety-five lepers, namely, those of Sherburne, near Durham, of Carlisle, and Bolton in Northumberland. Bloomefield, in his History of Norfolk, states that there were eighteen lazarhouses in Norfolk alone. In some of these the patients were amply provided for.

Astruc, Bach, and our own historians, Fuller and Heron, supposed that leprosy was introduced by those who returned from the Crusades, and brought it from the east. But the author of the present paper remarks that, even allowing that the disease is contagious, and that the increased national intercourse of that period may have tended to propagate it, there is ample evidence of its having existed in Europe, and even as far west as England, before the Crusades. It lingered long in Scotland after it had disappeared in England, and long in the northern islands of the former kingdom, as in Shetland, after it had left the mainland. In the Faroe islands (the land nearest the Shetland, northward), and in Iceland, it either still exists or existed at a very late period. In 1768, Peterson found 280 lepers in hospitals in Iceland, Olafsen and Henderson described the disease as existing when they visited the island in 1818. And the French expedition of 1836 brought back coloured sketches of natives affected with tubercular leprosy, which sketches are now in course of publication. Dr. SIMPSON, Prof. of Midwifery in University of Edinburgh, *Edinburgh Med. and Surg. Journal, Oct.* 1, 1841.

CONNEXION OF CLIMATE WITH DISEASES OF MALARIAL ORIGIN. The author shows in a series of interesting statistical observations and tables, the dependence of intermittent and remittent fevers on climate and temperature: but that synocha, synochus, common continued, ephemeral, and inflammatory fevers are nearly or wholy dependent on these causes. Dysentery and diarrhœa, on the other hand, are remarkably influenced by them: bearing, in this respect, a striking analogy to intermittent and remittent fevers. He rejects the notion that colic and cholera are dependent on exalted temperature: and questions the opinion of British statistics as to the connexion of hepatic affections and tropical temperature. He distinguishes between miasmata and mere effluvia. The latter, which are the mere elements of a compound body, are as innocuous as the compound itself; but the former may be new compounds, "resulting from the play of affinities among those liberated atoms." He states it to be a fact well known in the United States and in Canada, that although cultivation ultimately renders a climate more salubrious, yet endemic diseases, for several years after the soil is first cleared, become more severe and often assume an epidemic character. This is owing to the surface of the soil being exposed to the sun's rays, and consequently yielding a more noxious and copious malaria than when protected by a dense vegetation.—Dr. FORRY, *American Journal of Medical Science*, No. iii. *July*, 1841.

[We need scarcely apprize the reader that Dr. Forry's observations apply to the American continent only.]

ON THE APPLICATION OF MATHEMATICS TO THE SCIENCE OF MEDICINE. [This is a sensible paper on the value of the numerical method in medical research: the advantages of which are fully pointed out in our Twenty-third Number, article first: every possible doubt as to its unequivocal utility and importance is set for ever at rest. We shall briefly quote an interesting illustration of the satisfactory results of the application of numbers, in medical practice, in the case of a disease, regarding the respective value of the different modes of treating which, it was supposed, nothing definite could be determined.]

During the prevalence of cholera in Limerick, one of the writers of this article was first attached to St. Munchin's Hospital, where he tried every mode of treatment suggested by the profession, with so little apparent success, that he lost all faith in the influence of medicine. In this frame of mind he was transferred to St. Michael's Hospital, where the admissions were very numerous and the mortality alarming. He there found the calomel practice pursued with much more boldness and resolution, than any results he had witnessed could have induced him to adopt. As it was little complicated with the use of opium or any other medicine which could materially interfere with its operation, he determined to become a mere observer of its results for one month, that he might obtain some numerical evidence of the good or evil which at least one powerful remedy was capable of effecting. To arrive at more accurate conclusions, he noted in each case, on admission, the presence or absence of the pulse at the wrist, and took care that the registrar or apothecary did the same whenever he was absent. He also, in conjunction with the other physicians, endeavoured to prevent as much as possible the admission of patients in the mere premonitory stage, which, however, was not always possible. At the termination of the month he found the gross amount of deaths was 47, out of 165 cases, or less than one third, which was not far away from the general mortality in most countries which had been visited by the disease. He had here a proof that the numerical method, if applied only to the admissions, general treatment, and gross results, could lead to no practical inferences; for as the proportionate mortality in St. Michael's Hospital was nearly the same with the mortality in every other hospital in the kingdom, he could deduce nothing either favorable or unfavorable to the treatment from any comparison. But his enquiry had a closer application, inasmuch as the patients were classified on admission into those not yet in collapse, or who had a perceptible pulse at the wrist, and those in collapse whose pulse was not perceptible. On casting up the tables and ascertaining the amount of mortality in these separate classes, he was perfectly astonished to find, that in the first class, affected with rice-water vomiting, and purging, and suppression of urine, there were only 5 deaths out of 119 cases, while there were 42 deaths out of 46 cases admitted in a state of collapse. He had here at once evidence sufficient to lead to the most certain and confident conclusions. Cholera, so far from being a disease difficult of cure, was obviously more readily and certainly brought under the control of powerful remedies than any other known malady of so formidable a nature, and failures occurred entirely from neglecting to adopt a sufficiently bold and energetic treatment in the early stage; the only one in which the system is susceptible of the action of remedies.

The same method of observation applied to ascertain the extent to which calomel should be employed in cholera, showed that "salivation was wholly unnecessary for the cure," and the authors are therefore perfectly warranted to draw the conclusion which they do. "These imperfect illustrations of the accurate results to which the numerical method may lead in medicine; and above all, in those diseases which have equally baffled the industry of the morbid anatomist, and the speculations of the most philosophic theorist, may suffice in some degree to show what might be accomplished by a perfect and well-considered application of it in the investigation of disease." Dr. Griffin, *Dublin Journal of Medical Science,* No. lix. *Nov.* 1841.

**Plague not Contagious.** On the 2d of December last, the Zebra was driven into Kaiffa, on the coast of Syria, and the crew having been landed, lived for some time in the town, which is described as abounding with all "manner of abominations." A short time after this, cases of plague occurred at Acre, in repairing the fortifications of which, part of the crew of the Zebra was employed. This detachment returned to the vessel on the 15th of February, on the 17th of the same month, the Castor arrived at Kaiffa with orders to break up the Zebra aud embark her crew; and on the 20th, fourteen men belonging to the latter vessel were sent on board the Castor, in exchange for a party of artificers. On the evening of this day, a seaman belonging to the Zebra, who had been employed in the boats at Acre, was attacked by what is called fever, but which we understand, from what follows, to have been plague; and between that day and the 25th, thirteen more cases were added; but whether all consisting of the party employed at Acre is not stated. The men were instantly removed on board the Castor, and the disease now manifested the character of genuine plague. In order to prevent the diffusion of the malady eleven men were selected to attend wholly on the infected persons. Not one of these was attacked by the disease, nor were any of the Castor's people, except an artificer who had been landed and lived with the Zebra's men at Kaiffa, though there were at least twenty-four persons, including four medical officers, fully exposed to the contagion during its continuance on board the Castor.—*From a communication to the Admiralty, by* SIR WILLIAM BURNETT.

HEREDITARY TENDENCY TO MENTAL DISEASE. The object of this excellent essay is strictly conformable to the motto, which consists of a quotation from Sir James Mackintosh's well-known letter to the late Rev. Robert Hall, of Bristol, on the recovery of the latter from an attack of insanity. "Whoever," writes Sir James, "has brought himself to consider a disease of the brain as differing only in degree from a disease of the lungs, has robbed it of that mysterious horror which forms its chief malignity." The author accordingly states it to be his object to divest "the subject of predisposition (to madness) of all the mystery with which it has been invested......and to show that it depends upon the physical structure of the brain and nervous system." He points out the tendency, in certain families, to certain forms of madness, dependent doubtless on a particular family organization. Where hereditary tendency exists, he thinks its non-development is to be referred to one of three causes: 1st, One of the parents has been untainted and of robust constitution; 2d, The natural physical power and training of the individual himself have counteracted the hereditary predisposition; 3d, Rank, position, or profession, have removed him from influences calculated to develope that disposition. In 76 out of 514 cases, the disease descended from the mother; in 57, from the father; in 30 cases, two relations were deranged, in 484, only one relation. Both the parents of 6 of the cases were mad. The character of these cases, which would be interesting, is not given. The author concludes with expressing his belief that "hereditary tendency to mental disease is dependent on organization, is subject to the ordinary laws of the system, and in so far as our knowledge of that organization and of these laws extends, may be cured, counteracted, and ameliorated, in the same way, and on the same principles, as the tendencies to other diseases."—Dr. BROWNE, of Crichton Institution, *Phrenological Journal*, Nos. xiii and xiv. *Sept.* and *Oct.*, 1841.

ON DIABETES. In this paper, which is the outline of an essay commenced several years ago, and which the recent publications of Dr. Prout prevent the author, who has paid much attention to the subject, from giving in a more extended form to the public, the views entertained of the pathology of the disease by various authors are briefly given, and the objections which may be urged against those views then stated. These we omit, but give the author's explanation of the phenomena of diabetes in his own words:

"The disease, except when caused by some local injury, is dependent upon an accumulation of carbon and oxygen, and a deficiency of nitrogen, in the system; whether owing to the quality of the ingesta or atmosphere, or to a morbid condition of one or several organs destined to eliminate carbon alone or combined with oxygen, i. e. the lungs, the liver, and, in a majority of cases, the skin: those principles being directed to the kidneys, which, in consequence, set up an increased and vicarious action; a considerable deviation of the nervous system from the normal state being, in some cases, the cause, in others, the necessary effect of those morbid phenomena."

In supporting these views, the author observes that the surplus of carbon and oxygen may be accounted for, 1st, From insufficient elimination of carbonic acid gas by the lungs, in consequence of structural disease of those organs or of any cause that diminishes their power of eliminating carbon, as a course of mercury, nitric acid, strong tea, depressing passions. 2d. From disease of the liver and consequent disturbance of its power of decarbonizing blood transmitted to it. 3d. From disordered function of the skin. 4th. From a diet chiefly or exclusively vegetable. 5th. "Carbon may accumulate in the system in consequence of its constant absorption from the atmosphere, which, in our populous manufacturing districts, is so abundantly charged with carbonaceous matter."

[An endeavour is made by alleged facts and reasonings, to substantiate the preceding views. The subject, with Dr. Stanley's views of it, is an extensive and difficult one, and not to be discussed on an occasion like the present. With regard to the absorption of carbonaceous matter from the atmosphere, as a cause of diabetes, we have only to remark that it is a mere and not a very probable conjecture. We are also inclined to believe that, if, under a vegetable diet, less carbon is given forth by the lungs, it is because less of that principle is generated in the system, and that therefore there is less to be eliminated.]—Dr. Stanley. *London Medical Gazette.* No. 50. *Sept.* 3, 1841.

On the Subjectivity of Disease. The following extracts contain the most tangible part of the author's views on certain matters, the difficulty and obscurity of which and our want of the necessary information, render *speculation* perhaps the only legitimate method, as yet, of treating the subject.

The process of self-maintenance taking place by means of the minute organisms (the cells), has for its conditions a continual self-procreation at all portions of the body, and which, in other words, is a constant individualising (that is, the formation of cells, possessing each inherent life of its own); but running parallel with this power and action is seen a resolution of the individual life into the general influence exercised by the totality or universal organism, and by which an ever restless change, or circle of organic activity, is kept up and preserved until death comes on.

Now, if all tissues, organs, and systems are composed of simple individualised cells, it will follow that the process of disease falls within the sphere of these minute organisms, and must be viewed as an anomalous form of cellular life or activity, which can no longer act in harmony with, nor is influenced by, the universal tendency before alluded to.

That in inflammatory affections an altered or diseasedly-excited formative action is present, is evident from their production of morbid growths, formation of membranes, hypertrophies, and so on; and it is true that this has been acknowledged, but these *after-productions* grow, and are evolved according to laws of formation, regulating the whole organism itself in its primordial development; namely, out of the cells, and their power of evolution: that these productions are just as much *after-lives*, as what we denominate healthy structures, proceeding from and produced by a special, ultimate, and objective mode of life in the cells, has not been so acknowledged until very lately, indeed, by a few German physiologists. Allowing such to be origin of the *evolution* of diseased life, it is evident that two strictly subjective conditions may be existing in an organized body at one and the same time, namely, health and disease; and, therefore, the latter is not a mere *negation*, but something *positive*. Diseased life develops itself, like healthy life, out of a nucleus, and has just as much an essence or substratum at the bottom as the other has, and is no more the negation of health than evil is the negation of good.

We would, therefore, agree with Stark and Sobernheim, that disease is not the *negation* of health, but is something *positive;* and that in the theory of cellular evolution is to be sought the origin of all pathological formations; that all forms of exudation, membranose and pseudo-membranose structures, tubercle, carcinoma, hydatids, and moles, &c., are ultimately dependent upon a peculiar objective form of cellular life, as well as obliterations of vessels, hypertrophies, and hepatizations.—Dr. Hughes Willshire, *Lancet,* No. iv. *Oct.* 23, 1841.

Presence of Sulpho-cyanogen in the Saliva. In this paper, the author, after stating that he will not bring forward any evidence to prove the presence of sulpho-cyanogen in the saliva, it being generally admitted that it is rarely absent in the perfectly healthy individual; and after quoting Dr. Ure's observation that the absence or presence of the substance may afford a valuable indication in doubtful cases, gives several cases, and an illustrative tabular view, and thinks himself warranted in drawing the following conclusions, with which his paper finishes:

1. That sulpho-cyanogen is frequently not present in the saliva secreted by patients labouring under various diseases, at least that it is not discoverable by the sesquichloride of iron.

2. That it is frequently absent in febrile diseases, and in others where the permanent range of the pulse is considerably above the natural standard.

3. That it is invariably absent in distinct salivation produced by mercury, and that the non-reaction of the sesquichloride of iron upon the saliva does not seem to depend upon its great dilution, for when concentrated the same indications are produced.

5. That the presence of certain foreign constituents in the saliva, such as sugar in diabetes, may probably prevent the action of the reagent, or in some cases cause the red colour to be evanescent.

6. That, though the presence of sulpho-cyanogen in the saliva is not to be depended on as a criterion of good health, yet that its absence ought to excite an investigation into this point.

7. That the absence of sulpho-cyanogen in the saliva, when taken separately, is no proof of the action of mercury on the system; although when this secretion is copious, fetid, of light specific gravity, attended by spongy or ulcerated gums or cheek, and accompanied with this chemical characteristic, there will exist the strongest reasons for believing that this mineral has been exhibited.—*By* W. DAVIDSON, M.D. Physician to the Glasgow Infirmary, *London Medical Gazette*, No. X. *Nov.* 26, 1841.

ON THE USE OF SETONS, ESPECIALLY IN THE TREATMENT OF PARAPLEGIA. In this paper the advantage of setons is illustrated by two or three cases of induration and tumefaction in the abdomen, the result apparently of acute and chronic peritonitis. "In a variety of cases," observes the author, "of acute or chronic, local or limited internal inflammation, I have had recourse to the seton, and uniformly with the most marked success. . . . . . Hepatitis and nephritis belong to them in an especial manner. . . . . . The efficacy of setons, when appropriately applied in the nucha, (for they are frequently employed very uselessly), is well known." But the case to which the author would particularly call attention is, that of inflammation and congestion of the spinal marrow, with paraplegia or paraplegic spasm. He insists strongly on the seton being inserted *above*, not below, or on a level with the apparent seat of the spinal affection; and expresses it as his conviction that counter-irritation applied along the spine has failed, because it has not been applied below the seat of the disease. He was consulted recently by a gentleman with partial loss of power and sensation in the lower extremities, and numbness extending to "a line just above the sacrum. Issues had been applied on each side of this line. They might, with equal efficacy, have been applied to the foot!"—Dr. M. HALL, *London and Edin. Journal of Med. Science.* No. xi. *Nov.* 1841.

[We think Dr. Hall exaggerates the importance of the rule which he lays down. The intercommunion of sanguineous, and nervous tissues, and the known mutual sympathy existing between the various parts of the spinal cord, make it seem at least immaterial whether any topical application, more especially of so chronic a character as a seton, be made an inch or two above or below the precise seat of lesion. How often do we see epispastics applied on the calves produce notable relief of cerebral affections?]

ON THE DROPSY, WHICH FOLLOWS SCARLATINA. An important fact noticed by the author of this paper, in every case examined by him, of fatal dropsy following scarlatina is, that the effused fluid contained a notable portion of urea. He has tested fluid from the ventricles, pericardium, pleuræ, peritoneum; and in every case he found urea. In all the cases treated by him during the last four years amounting to forty or fifty, the kidney was affected. The results of his own experience, consequently, had led him to conclude that this organ invariably suffered; but the fact of Dr. Phillip having had sixty cases in Berlin, in none of which the urine, tested by heat and nitric acid, betrayed a trace of albumen, has led him to modify that opinion.

If the author would be disposed to admit, under any circumstances, the existence of an idiopathic and general inflammatory state, apart from any local affection, it would be in acute dropsy succeeding scarlatina. Although, in all his own fatal cases, the kidneys were found affected, he does not think it by any means certain that the disease began in these organs; and he holds it to be very questionable whether the state of the kidney had anything to do with the general disease.

The author thinks the dropsical symptoms consequent on the discharge of albuminous urine easily explicable, by supposing that the blood, being drained of its albumen, permeates its vessels, and infiltrates into the cellular tissues.—Dr. Robert Willis, *in London and Edin. Journal of Medical Science*, No. x.

On some Diseases in which Albuminous Urine occurs. In this paper, somewhat akin to the preceding, the author points out that Cotunnius, Fordyce, and Rollo were cognisant of the occasional connexion of anasarca and albuminous urine. This sort of urine may manifest itself in the course of several diseases, in which there is not a trace of granular or other degeneration of the kidney. It has been observed in pneumonia, pertussis, diabetes insipidus, icterus, chronic bronchitis, and diseased heart, in rheumatism, in modified variola. In some of these cases, examined *post mortem*, no structural change in the kidney was detected; and the author states that, in the other cases, there was no ground for suspecting such a change. He is of opinion that, between renal and scarlatinous dropsy, and between the lesion of the kidney which characterizes scarlatina and Bright's disease respectively, there is an essential distinction. He considers the unusually low specific gravity of the urine which accompanies granular degeneration of the kidney, much more important, as a single diagnostic sign, than the presence of albumen.—Dr. Williamson, *Edin. Medical and Surgical Journal, Oct.* 1841.

On the Signification and Ends of the Portal Circulation. The object of this paper is to show that the portal circulation is contrived with a view to "economise arterial blood." The author observes that "the supply of so large a viscus as the liver with arterial blood, for a purpose which, however important in itself, is still only secondary, would have been a serious matter, and would have implied an increase in the pulmonary system especially, to an extent which must have been felt as inconvenient. Had the liver been furnished with arterial blood, as the means of affording bile, it must obviously have had a vessel sent to it, of a caliber equal to the sum of the vessels whose contents are finally collected into the portal vein. Nature goes more economically to work; she first uses the blood of the great abdominal arteries to vitalize the viscera, and then, gathering this into a common trunk, she sends it to minister to the function of the liver; this blood, as a vivifying fluid, is exhausted for the time, but it will still yield the elements of bile, if subjected to the elective affinity of the liver. The whole system of the liver is, in fact, calculated on economic principles, &c."—Dr. Robert Willis, *Lond. and Edin. Month. Jour. of Med. Science*, No. ix. *Sept.* 1841.

[The interesting nature of the question considered in this paper, and the importance we attach to the opinions of its author, induce us to make a few remarks on it.—Without wishing to deny the general correctness of the author's view, we may remark that it appears to us far too exclusive. In carrying out his speculation, Dr. Willis has neglected several elements of great importance. His statement that "there can be nothing in the nature of the blood of the portal system, which fits it more than any other for furnishing the elements of bile," and that "individuals have lived and thriven without the portal system," is not consistent with what we deem the best physiological view of the subject. We are not aware of any instance, in which individuals have lived to adult age with such a malformation as that alluded to; and in the only case (that recorded by Mr. Abernethy,) in which the liver was examined with sufficient care to enable a decisive opinion to be given, it was clear that a considerable amount of venous blood might have circulated through the organ. Moreover, it seems probable, that, even if the hepatic artery furnished the only supply in such instances, the blood would have become venous before passing into the secreting capillaries. The former part of the assertion is hazarded incautiously: it is well known that the blood returning from the intestines is charged with a large quantity of crude materials absorbed from their contents: in the invertebrate animals the mesenteric veins are the only absorbents; and even where a special system (the lacteal) is evolved, it has been clearly demonstrated that they still retain, in a great degree, their original function. Now there is good reason to believe that a portion of these crude materials is eliminated by the liver, so that the blood returning from the intestines undergoes a purification before it enters the general current of the circulation; in the same manner as the blood which has received the chyle, is submitted to the action of the lungs before it passes again to the system. In cases where the portal system has been deficient, (even supposing the deficiency to be complete,) the arterial blood will become *biliferous*, (so to speak,) and will therefore afford, though in a less concentrated form, the elements of the secretion. The great size of the liver in many of the lower classes of animals, relatively to the respiratory apparatus, shows that it is in them the most important organ for depurating the blood; and, if blood were to pass to it from the arterial system, it would be no more *arterial* in its character, than is that of the pulmonary artery in air-breathing vertebrata.]

ON HIPPURIC ACID AND ITS TESTS. IN a paper communicated by me to the Medico-chirurgical Society in the month of January last, it was pointed out for the first time, that when a certain portion of benzoic acid, or of a soluble benzoic salt, is introduced into the human stomach, a remarkable change takes place in its passage through the kidney. The urine voided in the course of a couple of hours after its ingestion, amounting usually to five or six ounces, will be found, upon adding a twelfth part of muriatic acid, to yield by and by a copious precipitate of beautiful rose-pink acicular crystals. These, when examined by the microscope, exhibit the form of a four-sided prism, terminated by a dihedral summit. Now this is precisely the crystalline character of an acid peculiar to the urine of the horse, cow, and other graminivorous animals, and to which, for that reason, Liebig has assigned the name of *hippuric*.

By this singular interchange of elements, capable of being effected only by the aid of vital chemistry, we have an organic product, uric acid, containing eight atoms of azote, and ten of carbon, replaced by one, hippuric acid, containing no less than eighteen of carbon, and only two of azote! In pursuing the above investigation a step further, it was ascertained that no trace whatever of uric acid or any of its salts could be discovered in the urine in question. In point of fact it had been wholly superseded by the other acid.

The important circumstance connected with this research, as bearing upon medical practice, is that the salts which this new acid forms with the ordinary bases occurring in the animal fluids, as soda, ammonia, and potash, are all of easy solubility. Thus hippurate of soda requires about two parts of water, at 60° Fahr. to dissolve it, whereas the corresponding uric salt, which constitutes, as is well known, the gouty concretions or chalk-stones, is acknowledged to be nearly as insoluble as uric acid; it requires at least 4000 parts of water to dissolve one. Hippurate of ammonia again is but little less soluble than hippurate of soda, while urate of ammonia will only dissolve to the amount of 1-480th part. Hippurate of lime, the least soluble of these salts examined by me, requires 18 parts of water to dissolve one.

The application of the above principle has proved of material benefit in the treatment of certain unhealthy conditions of the urine, occurring in subjects of a calculous or gouty diathesis; since it enables the practitioner to obviate entirely the various deposition resulting from excess of uric acid, the fruitful source of that most distressing malady, stone in the bladder; as also to control and prevent the formation of the so-called tophaceous concretions or chalk-stones, which occasion so much inconvenience, deformity, and pain to individuals labouring under gout.

By judicious exhibition of the benzoic acid, or, according to particular circumstances of a benzoic salt, that is to say, apportioning the dose to the state of the renal secretion, best previously ascertained by analysis, we can fulfil the desired indication with unerring precision, and that, as shown in the above paper, without any risk of affecting the general health, or of irritating the urinary organs.

It is to be kept in mind that this plan of treatment by no means precludes the adoption of other suitable remedial measures. Of course certain rules of diet must be observed, which there is no necessity for referring to here. It would be equally irrelevant to occupy your time with the details of cases, several of which have lately come under my notice, and go to corroborate the statements above made.

As benzoic acid is apt to irritate the fauces, unless administered in a liquid state, and as it needs a large quantity of water to dissolve it, it will be found expedient to give it along with phosphate or biborate of soda; since they enhance its solubility without abating its specific power. Thus, four parts, by weight, of the former, or one part and a half of the latter salt, will enable a comparatively small proportion of distilled water to take up one of the acid. This difficulty, as is obvious, does not apply to the benzoate of ammonia or of potash.

Phosphate of soda not only serves to hold benzoic acid, but likewise hippuric acid, in solution; and this is a point of some consequence, seeing that any excess of the latter acid, accidentally present in the urine, will remain dissolved by means of the neutral phosphate of soda, or the triple phosphate of soda and ammonia (microcosmic salt), naturally existing in that secretion. These phosphates, however, produce a very different effect upon the uric acid, inasmuch as they promptly convert it into urate of soda, by depriving the salt of one-half of its base, and thereby transforming it into biphosphate. This fact, which recently presented itself to me in the course of some experiments, and which has not been heretofore noticed by any chemical authority, seems to furnish a simple and rational explanation of the mode of formation of urate of soda, the basis of chalk-stones. Hence whenever the oxidizing process of the kidney is proceeding with such energy as to supply a superabundance of soluble phosphates on the one hand, and of uric acid on the other, there must inevitably result an excess of urate of soda.—Alexander Ure, M.D., A.M., *Pharmaceutical Transactions, July* 1, 1841.

